# Feasibility of the Social Media–Based Prevention Program “Leduin” for German Adolescents on Instagram: Mixed Methods Pilot Study

**DOI:** 10.2196/78774

**Published:** 2025-11-27

**Authors:** Elizabeth Zimmermann, Samuel Tomczyk

**Affiliations:** 1 Department Health and Prevention, Institute of Psychology, University of Greifswald, Robert-Blum-Str. 13, Greifswald, 17489, Germany, 49 3834420 ext 3807; 2 German Center for Child and Adolescent Health (DZKJ), Partner Site Greifswald/Rostock, Greifswald, Germany

**Keywords:** social media, adolescents, feasibility, intervention, life skills, mixed methods, user engagement

## Abstract

**Background:**

Digital platforms, particularly social media, including Instagram, present unique opportunities for health promotion among adolescents due to their widespread use with interactive features supporting high user engagement. However, the feasibility of effectively utilizing platforms like Instagram for health interventions requires careful consideration of adolescent engagement patterns.

**Objective:**

This pilot study evaluated the leduin program—designed to foster essential life skills and functional social media use among adolescents—while also exploring the broader feasibility of using Instagram to deliver complex social and psychological interventions in this population.

**Methods:**

The study adapted the feasibility framework by Bowen et al and used a mixed methods approach. Quantitatively, Instagram interaction metrics of 99 participants (women: 62/99, 63%; men: 37/99, 37%; aged 14‐18 years; mean age 15.2, SD 0.74 years) were analyzed descriptively (means, medians, SDs) and inferentially (Welch ANOVA, Kruskal-Wallis, Pearson and Spearman correlations, linear and segmented regression analyses) using RStudio. Metrics included story views, retention rates, feature engagement (eg, polls, question stickers, quizzes), and drop-off rates. Recruitment efforts were also analyzed descriptively. Qualitatively, 13 postprogram semistructured interviews were conducted with 11 women (11/13, 65%) and 6 men (6/13, 35%; mean age 15.29, SD 0.99 years). Participants were sampled to reflect varying engagement levels (6 high, 5 medium, 6 low). The mean interview duration was 25 minutes 11 seconds (SD 6 minutes 34 seconds). Content analysis, with high intercoder reliability (κ=0.90), comprehensively explored participants’ experiences and the program’s impact.

**Results:**

Quantitative results indicated that the recruitment process was challenging, with 101 schools and 10 youth centers contacted, resulting in a participation rate of 12.8% (99/775 students). On Instagram, story views ranged from 34 to 81 per post, with an average daily retention rate of 87.7% (SD 7.8%). By week 4, 76% of the total drop in views had occurred (mean views declined from 66.1 to 53.4); by week 6, 97.3% of the drop had been reached (declined from 66.1 to 49.9 views), indicating sustained viewer interest over the 14-week program. Features requiring minimal user effort, including polls (mean 56.8%‐54.4%), quizzes (mean 56.6%), and sliders (mean 51.2%), showed significantly higher interaction rates than more demanding features such as challenges (mean 21.7%) and question stickers (mean 20.6%; *P*<.001). Qualitative findings revealed that adolescents valued the program, its design and methods for its relevance to their daily lives, and its support in developing essential life skills. Suggestions for improvements were made.

**Conclusions:**

The study underlines the potential of various Instagram features and content posting schedules for health interventions to meet adolescent preferences and interests. Challenges with reaching the target group effectively emphasize the need for targeted recruitment strategies and optimizing initial content to boost engagement, underscoring the critical implications for prevention research and policy in leveraging digital platforms to enhance adolescent health.

## Introduction

### Opportunities, Risks, and Skills in Adolescent Social Media Use

Digitalization has profoundly transformed modern culture, by blending physical and virtual worlds. Consequently, how individuals communicate and engage with information has been reshaped. A central element of this shift is the rise of social media, which now connects more than 5 billion users worldwide [1]. Adolescents, defined by the World Health Organization (WHO) as individuals aged 10 years to 19 years [2], are among the most active social media users [3]. Accordingly, they are particularly affected by the dual nature it presents. On one hand, platforms like Instagram, YouTube, and TikTok enable the creation and sharing of content on an unprecedented scale, facilitating social interaction, identity exploration, and self-expression [4-7]. In doing so, social media can support key developmental tasks for adolescents such as building autonomy, self-efficacy, and social capital while also providing representation and empowerment for marginalized groups [6,8-10]. On the other hand, social media entails risks including misinformation, privacy risks, and the need for critical consumption skills [11,12]. Due to their still-developing cognitive, emotional, and digital competencies, adolescents are highly vulnerable to risks such as exposure to harmful content, cyberbullying, and platform mechanisms that may support addiction [13-16]. Accordingly, problematic social media use—characterized by preoccupation and compulsion—has been linked to mental health issues, including depression, anxiety, and stress [17-19]. This highlights the need for the development of skills to functionally use social media [15,20].

### The Role of Social Media in Promoting Adolescent Health

Although social media poses benefits and risks, it also presents unique opportunities for health promotion and prevention: Its ability to reach adolescents within their daily routines—among teens aged 13 years to 17 years, YouTube is used by 90%, TikTok is used by 63%, and Instagram is used by 61% [3]—makes it a low-threshold, cost-effective, accessible, and engaging medium for interventions [21-25]. For example, a recent meta-analysis found that social media recruitment nearly doubled cost-efficiency over traditional methods and outperformed other online approaches by 66% [23].

Under these favorable conditions, social media interventions have effectively promoted health behaviors among adolescents: Reported successes include improving physical activity and diet, increasing safe sexual practices, raising mental health awareness, and promoting skin health with a small but significant overall effect size of a Hedges *g* of 0.24 [26-28]. These findings indicate the potential of social media as a tool for health promotion, though effectiveness varies by health topic and study.

### Interactivity and User Engagement

Interactive elements play a crucial role in social media interactions: Research underscores that interactivity boosts engagement, symptomatic improvements, and learning outcomes [29] by promoting a feeling of connectedness with the program [30]. Interactive features such as social forums, peer interaction, and gamification elements including challenges and rewards significantly enhance user involvement, reduce dropout rates, and encourage behavior change [31-37]. Additionally, effective program design that utilizes multimedia, regular interaction by hosts, and relevant links supports sustained user engagement [37,38].

Instagram, known for its rich interactive features, is increasingly being researched as an effective platform for health promotion [39-42]. It offers significantly higher interaction rates than platforms like Facebook or Twitter, with interaction rates 30 to 200 times higher in Instagram-based health campaigns [43]. The possibilities of the Instagram story continue to evolve, incorporating advanced features such as polls, quizzes, shopping links, countdowns, and swipe-up links. These stories can be saved beyond the typical 24-hour period using the “Highlights” feature, enhancing their utility for prolonged engagement [44]. Recent research underscores the importance of using relatable visuals, authentic language, humor, and culturally resonant messaging to align with the audience’s social and cultural identities for effective health promotion on Instagram [45-47]. Further, the necessity of addressing barriers such as digital literacy and privacy concerns and incorporating interactive elements to boost the accessibility and effectiveness of these interventions have been highlighted [48].

Despite these promising developments, however, evidence remains limited on how to use Instagram to deliver complex social and psychological interventions, since most existing interventions focus on single behaviors or brief interventions. To address this gap, we initiated a broader research project to explore how Instagram can be used as a delivery platform for structured and complex, interactive interventions (such as the leduin program) [49]. This paper focused on one component of that project: the feasibility evaluation of the leduin program. It built on the feasibility framework by Bowen et al [50], which has been widely applied across diverse health and digital intervention research fields [51-57].

Feasibility studies aim to assess whether the development and evaluation of an intervention can be realistically undertaken and whether it should proceed to further testing. They provide insights into implementation strategies and inform the design of larger trials [50,58,59]. Specifically, feasibility studies help determine the conditions under which an intervention might work prior to testing its actual effectiveness [60].

### Social Media Feasibility Studies

Existing research provides mixed but instructive evidence on the feasibility of delivering interventions via Instagram. Some studies highlight the platform’s potential to reach adolescents effectively: For instance, Instagram ads recruited a diverse adolescent sample with over 96% retention at the 16-week follow-up [61], and brief video-based interventions targeting depression and anxiety symptoms were successfully delivered to youth [62]. Additionally, social media metrics have proven to be a practical tool for measuring engagement [46].

However, other findings underscore substantial limitations. In a human-centered outreach campaign, fewer than 25% of adolescents followed the Instagram account after 2 months, and only 7% of followers matched the intended target group [47]. A 12-week exercise intervention for young women showed high satisfaction but only 56% retention and low active engagement—just 30% consistently liked posts, and few commented or tagged others [63]. Similarly, a 13-week physical activity program reported good reception but poor survey completion rates, dropping from 33.3% at 4 weeks to 22.2% after 3 months [64].

### The Leduin Program

Recognizing both the opportunities and risks of social media, we aimed to harness its potential while addressing its challenges. Accordingly, we developed the *leduin* program (short for “Lebenskompetent durch Instagram,” meaning “Life skills through Instagram”) for adolescents delivered via Instagram. It teaches life skills for use in both analog and digital settings (see Zimmermann and Tomczyk [65] for examples). It draws on the Ottawa Charter for Health Promotion [66] and the definition of life skills as outlined in the WHO’s life skills framework [67], including decision-making and problem-solving, creative and critical thinking, communication and interpersonal skills, self-awareness and empathy, and coping with emotions and stress. In sum, these are fundamental skills to navigate the challenges of everyday life [15,16].

**Figure 1.**
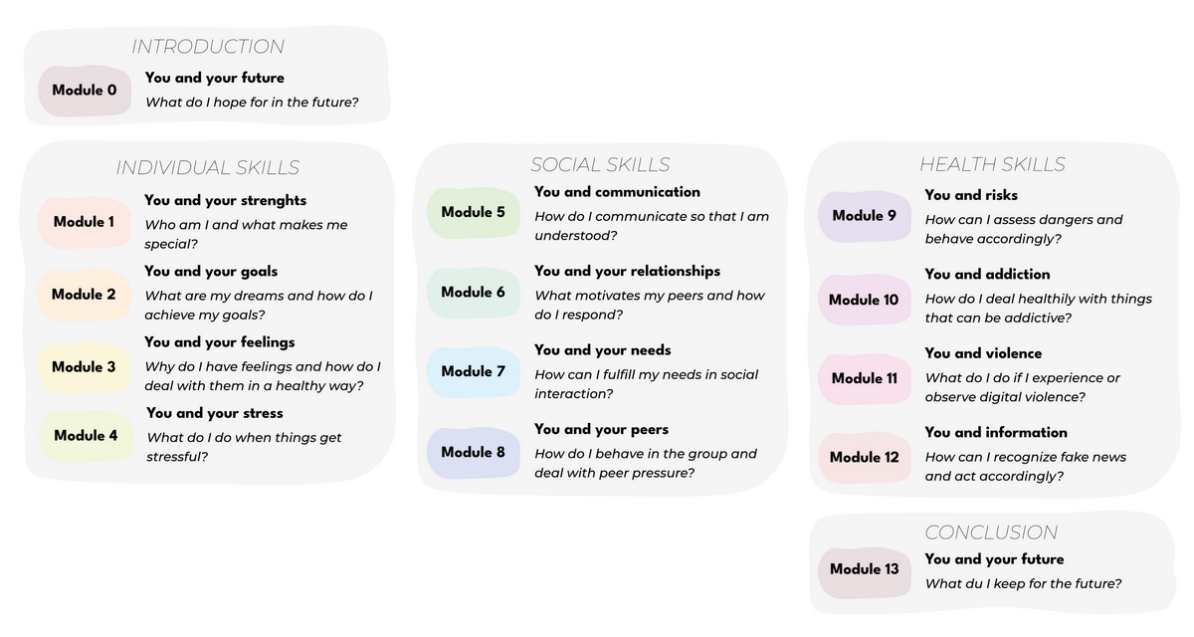
Module structure of the 14-week life skills program leduin.

The development of the leduin program preceded this feasibility study. It followed the intervention mapping framework [68] and drew on established psychological theories as well as co-creation to guide content and structure (see Multimedia Appendix 1). Adolescent perspectives on program design and Instagram features guided the co-creation process [69]. The intervention lasts 14 weeks and includes weekly modules addressing individual, social, and health-related skills as shown in Figure 1. Each module introduces a real-world challenge and links it to digital life skills. It is delivered through Instagram stories and feed posts aligning with WHO life skills education guidelines [70]. The program is conveyed via a private Instagram account. Only adolescents who actively decided to participate in the program are accepted as followers, creating a safe space and relevant social comparison among peers. Features such as polls, sliders, question stickers, and quizzes enable active participation. Weekly self-care tasks, journaling activities (via an optional analog workbook), and interactive challenges with raffles are included. These also promote sustained engagement and spaced learning [71].

In sum, to the best of our knowledge, the leduin program is the first prevention initiative to be co-created and delivered entirely on Instagram, designed to strengthen both life skills and social media skills in adolescents. It provides an exclusive environment for this age group and leverages Instagram’s interactive features to facilitate anonymous peer exchange and deliver multimedia content. The modular structure offers daily inputs, reflection tasks, and challenges, with learning seamlessly integrated into daily life and habits, thereby supporting behavior change.

### Objectives

The aim of this study was to evaluate the feasibility of delivering a complex, theory-driven life skills intervention for adolescents via Instagram using an iterative process with active involvement of the target group [72]. As part of a broader research initiative exploring the use of social media for digital prevention, this pilot study focused on the leduin program—a 14-week intervention designed to promote digital life skills in adolescents through interactive, platform-native content.

## Methods

### Study Design and Reporting Standards

This paper was written in accordance with APA Style Mixed Methods Article Reporting Standards (MMARS) including the Journal Article Reporting Standards for Qualitative Research (JARS-Qual) and Quantitative Research (JARS-Quan) [73,74]. We further used the Consolidated Criteria for Reporting Qualitative Research (COREQ) [75].

### Research Design and Theoretical Framework

This study used a pragmatic mixed methods approach, integrating both quantitative and qualitative data to provide a comprehensive analysis of the program [76]. Quantitative data were derived from Instagram usage metrics, directly reflecting participant behavior on the platform throughout the program. Qualitative data were gathered through interviews with program participants after program end. These interviews were analyzed using qualitative content analysis to deeply explore adolescents’ perceptions of the program [77]. The qualitative content analysis was guided by the feasibility framework by Bowen et al [50], which comprises 8 key domains for evaluating whether and how an intervention can work: acceptability, demand, implementation, practicality, adaptation, integration, limited efficacy testing, and expansion potential.

The unique nature of a social media–facilitated intervention required adaptations to this framework to reflect the specific conditions of digital delivery. Unlike traditional interventions, Instagram-based programs are centrally administered and do not rely on repeated implementation by local practitioners. Although traditional interventions often depend on face-to-face facilitation and organizational infrastructure, social media interventions are disseminated digitally, engage users asynchronously, and can also be evaluated through platform-based engagement metrics. In the adapted version of the framework by Bowen et al [50] used in this study, the core meaning of each domain was retained but reinterpreted to align with the Instagram delivery format (see Table 1). Please see Multimedia Appendix 2 for more details.

**Table 1.** Overview of the feasibility framework by Bowen et al [50], including the domains and their adaptation for the Instagram-facilitated life skills program leduin.

Domain	What the domain evaluates	How it can be assessed
Acceptability	How well the target audience receives the intervention methods and design	Participant feedback on the program usability; engagement metrics of various features
Demand	Evidence of interest and participation by the target population of the intervention content	Participant feedback on the content and its relevance to their lives; engagement metrics on usage over time and the demand of different topics
Implementation	Whether the digital program delivery met adolescents’ expectations and when engagement declined during the intervention	Participant feedback on expectations; analysis of drop-offs across the intervention period
Practicality	Feasibility of integrating the program into adolescents’ daily routines	Participant feedback on time investment and use; quantitative weekday/view analysis
Adaptation	Flexibility of the intervention to meet target group needs or platform requirements	Considered conceptually; program intentionally fixed
Integration	Fit with existing social and institutional structures	Evaluation of the recruitment process to motivate program participation
Limited efficacy testing	Preliminary evidence that the intervention produces desired outcomes	Participants’ subjective learning experiences and perceived changes in behavior
Expansion	Scalability of the intervention to other groups or contexts	Considered conceptually; scalability inferred from platform reach and modular program structure; calculation of staff hours and cost/participant

### Ethical Considerations

The study was approved by the Ethics Committee at University Medicine Greifswald (BB 060/22). Approved activities included implementing the 14-week prevention program leduin on Instagram and conducting subsequent interviews with the participants postprogram.

All participants and their guardians were fully informed about the study’s objectives, methodologies, and data protection measures. To guarantee voluntary participation, informed consent was obtained within the school setting, with consent forms collected by the teachers accompanying the program. These forms were completed and submitted before the beginning of the leduin program and subsequent interviews.

Data collection and storage were handled in strict compliance with German data protection laws, ensuring the anonymity of all data to protect participant privacy. The publication omits all personal identifiers, maintaining participant confidentiality throughout the study.

There was no financial compensation nor other incentives offered to participants for their involvement in the study. Recruitment aimed to foster genuine interest and engagement in the program. Participation in certain more demanding challenges was encouraged by the opportunity to win prizes in a raffle.

No photographs of participants were captured. Accordingly, images are absent from both the manuscript and any supplementary materials. Consequently, no separate consent for images was necessary.

### Research Flow and Team

The leduin program began in October 2022 and ran for 14 weeks, with a 2-week break over the Christmas holidays, concluding in February 2023. Usage data were collected throughout, and interviews were conducted from February 2023 to April 2023 by 4 female researchers with psychology backgrounds, all affiliated with the University of Greifswald. The team included 2 undergraduate students and 2 students with bachelor’s degrees, part of a research group studying social media’s impact on health care.

These researchers were closely involved in the planning and implementation of the leduin program, which provided valuable contextual knowledge for the evaluation. At the same time, the team was aware of their dual role as program implementers and evaluators and the potential influence this could have on data collection and interpretation. To mitigate bias and minimize power dynamics, especially between adult researchers and adolescent participants, the main account holder—the visible figure during the program—did not conduct any interviews. Furthermore, the research team engaged in regular debriefings and reflexive discussions throughout the study to reflect on their positionality, discuss ethical considerations, train communication styles with adolescents, and critically examine how their roles and assumptions might shape interactions with participants and the analysis of data.

### Participants and Recruitment

Our study used a purposive sampling method, targeting adolescents in 9th and 10th grades aged 14 years to 18 years across schools in North Rhine-Westphalia and youth centers throughout Germany. This demographic was chosen because it represents a critical phase of development: Social norms strongly shape behavior and healthy habits formed during this time often persist into adulthood. Moreover, these grades are especially important as they prepare students for the transition to secondary education or graduation, highlighting their significance for timely intervention efforts.

To facilitate recruitment, the secretariats of several schools and youth centers were contacted via phone and email to engage teachers and social workers interested in implementing our program. Although the program was delivered primarily through Instagram, teachers and social workers played a key organizational role in recruiting participants. Additionally, they received weekly newsletters on program content and participant engagement, along with optional teaching materials for each module block.

Information sessions were conducted with interested classes and youth groups to thoroughly explain the program’s objectives, methods, and data collection procedures. Adolescents willing to participate had to return a jointly signed consent form that included their own informed assent and the written consent of their legal guardians, submitted via their educational or social work facilitators to the leduin team. Participation was restricted to those who were at least 14 years old and proficient in German to ensure comprehension of the program content.

In sum, 99 adolescents took part in the program: 62 (63%) identified as women, and 37 (37%) identified as men. The mean age was 15.2 (SD 0.74) years, ranging from 14 years to 18 years. Adolescents came from highly diverse transnational family contexts. Of the 99 participants, 60 (61%) had a migration background: 50 (50/99, 51%) had both parents born outside of Germany, and 10 (10/99, 10%) had 1 parent born abroad. The remaining 39 participants (39/99, 39%) had no migration background; both parents were born in Germany. Among those with a migration background, the most frequently reported countries of parental origin were Kosovo (mentioned 16 times), Ukraine (11 times), Morocco (11 times), Serbia (10 times), and Russia (5 times). Other countries mentioned more than once included Kazakhstan, Pakistan, Armenia, Iran, and the Netherlands. In total, parents were reported to have been born in over 25 different countries, covering regions across Eastern and Southeastern Europe, Central and South Asia, North Africa, and Western Europe. Of the 99 participating adolescents, 10 (10%) were recruited via a youth center and 89 (90%) via 6 different schools. These included 4 secondary schools (63/99, 64%) and 2 grammar schools (26/99, 26%).

### Data Collection

#### Quantitative Data Collection

Quantitative data were collected on both the recruitment process and program engagement. During recruitment, the research team documented process indicators, including the number of schools and youth centers contacted, participation in information events, and the number of students who voluntarily enrolled in the program. Throughout the 14-week intervention, 2 trained researchers manually recorded Instagram usage data in structured Excel (Microsoft Corp) sheets. This approach was necessary because the intervention was delivered via a private Instagram account, which limited access to Instagram’s native analytics features. We deliberately prioritized this privacy setting due to ethical considerations: The program was designed to be accessible only to adolescents who had actively consented to participation, along with consent from their caregivers. The private account ensured that only enrolled participants could view and interact with the content. This setup also guaranteed that peer exchange remained confined to the target group, with no possibility for teachers, parents, or external observers to access posts or responses. This privacy was essential to fostering a sense of trust, safety, and openness, especially since many of the program topics touched on emotionally sensitive or personally relevant issues. To address the limitations introduced by manual tracking, 2 trained researchers jointly recorded all engagement data using a standardized protocol to ensure accuracy and consistency. The resulting data informed the evaluation of 4 feasibility domains: acceptability, demand, implementation, and practicality (see Table 2 for an overview).

**Table 2.** Instagram usage data reflecting the interactions of participants with the program content and relevant feasibility framework domains.

Data	What the data say	Additional information	Relevant to evaluate the following domain(s)
Follower amount	Number of users who followed the Instagram account on a given day	Participants could join or unfollow the private account throughout the intervention due to organizational flexibility.	Integration
Module	Weekly module number (0‐13)	Each week addressed a different life skills topic.	Practicality
Day of the week	Day on which content was published	Content was distributed daily, from Monday through Sunday.	Practicality
Story views	Number of views each story slide received	Stories consisted of multiple slides, each with unique content.	All
Quizzes	Number of quiz responses and number of correct answers	Multiple-choice quizzes were related to each module’s content.	Acceptability
Polls	Number of votes submitted in polls	Polls allowed anonymous voting on module-relevant questions.	Acceptability
Poll by team	Number of responses to polls posted by the leduin team	Polls were created and shared by the program team.	Acceptability
Poll by users	Number of responses to polls based on peer answers	Participants’ anonymous answers were shared, and peers could vote on shared experiences.	Acceptability
Poll for challenge	Number of responses to polls asking for preferred challenge rewards	Polls asked participants to choose among possible raffle rewards.	Acceptability
Question stickers	Number of short answers submitted via question stickers	These were used to collect open-text responses to prompts or questions.	Acceptability
Challenge	Number of challenge submissions received	Participants edited screenshots of story slides and submitted them via direct message.	Acceptability
Slider	Number of times the slider feature was used	Sliders were used for self-assessment of skills or mood.	Acceptability

#### Qualitative Data Collection

Qualitative data were collected through semistructured interviews conducted after the conclusion of the leduin program. The primary objective was to gather adolescents’ perspectives to inform the feasibility evaluation and guide further program refinement. Interviews were conducted individually or in a group setting and followed ethical procedures. These included verbal reminders of participants’ rights to voluntary participation, withdrawal at any time without consequences, and assured confidentiality.

To capture a range of experiences, participants were purposively sampled based on their level of engagement with the program’s interactive challenges: high (more than 5 participations), medium (1 to 5 participations), and low or no participation. Only adolescents with prior consent for follow-up were contacted via direct Instagram messages. In total, 13 individual interviews were conducted: 8 orally via video conferencing (audio recorded and transcribed) and 4 in written form via Instagram direct messages. One group interview with 5 adolescents (3 women, 2 men) was held at a youth center, organized by a social worker. Despite efforts to balance gender representation, the final sample consisted of 11 women and 6 men (see Table 3 for an overview). Participants’ ages ranged from 14 years to 18 years (mean 15.29, SD 0.99 years). Engagement levels varied: 6 participants showed high engagement, 5 had medium engagement, and 6 had low engagement. The interviews lasted on average 25 minutes and 11 seconds (SD 6 minutes and 34 seconds; see Table 3 for an overview).

**Table 3.** Participants of semistructured interviews with 17 adolescents (11 women, 6 men) aged 14 years through 18 years with different engagement levels during the leduin program.

Interview	Type	Duration (minutes)	Gender	Age (years)	Level of engagement
01	Oral	20:29	Woman	14	High
02	Oral	24:38	Man	15	High
03	Oral	21:13	Woman	15	High
04	Oral	34:51	Woman	15	Medium
05	Oral	21:35	Man	16	Medium
06	Oral	14:46	Woman	14	High
07	Oral	25:20	Woman	16	High
08	Oral	31:43	Man	15	Medium
09	Written	N/A^a^	Man	15	Medium
10	Written	N/A	Woman	15	Low
11	Written	N/A	Woman	15	High
12	Written	N/A	Woman	16	Low
13	Group video conference	32:12	3 women, 2 men	Women: 16, 16, 18; men: 14, 15	Women: low, medium, low; men: low, low

a N/A: not applicable.

Interviews were guided by a semistructured protocol designed according to the recommendations for qualitative research with adolescents, allowing for both flexibility and structured inquiry [78,79]. This approach allowed spontaneous questions and topic shifts, fostering authentic dialogue and aiding the evaluation of complex subjects [80]. Interviewers could adapt to participants’ responses, ask clarifying questions, and pursue emerging themes, which supported the collection of authentic and context-rich data.

The interview guidelines focused on 5 of the 8 feasibility domains by Bowen et al [50] relevant to adolescent perspectives: acceptance, demand, implementation, practicality, and limited efficacy testing. Questions were designed to align with the theoretical framework while remaining accessible to adolescent participants. An overview of interview topics and their corresponding feasibility domains can be found in Multimedia Appendix 3, and the corresponding interview guide can be found in Multimedia Appendix 4.

Individual interviews were conducted online in private one-on-one settings to protect confidentiality. Adolescents joined the interviews from home. In the session held at a youth center, a social worker was also present to provide supervision, adhering to the protocols for conducting research with minors.

To enhance authenticity and reduce social desirability bias, all interviewers received intensive training based on the methodological approach to interviewing in social work by Widulle [81] and Reinders’ [78] recommendations for conducting qualitative research with adolescents [82]. Training emphasized relationship-sensitive interviewing, active listening, conversational warm-up techniques, mirroring responses, and empathetic communication to build trust and promote open dialogue.

Audio recordings were transcribed according to the guidelines by Dresing and Pehl [83]. Due to logistical constraints, transcripts were not returned to participants for verification. All materials used or generated in the qualitative phase, including anonymized transcripts and the coding framework, are available in the Open Science Framework (OSF) online repository [84].

### Data Analysis

To evaluate the feasibility of the leduin program, we applied a mixed methods design guided by the adapted framework by Bowen et al [50]. Quantitative analyses of Instagram usage data addressed 6 of the 8 framework domains, while qualitative data from semistructured interviews provided in-depth insights into 5 domains. Together, these complementary approaches enabled a comprehensive assessment of the program’s delivery, engagement, relevance, and perceived impact.

#### Quantitative Data Analysis

Quantitative analyses were conducted in RStudio (Posit Software, PBC) using R (Version 2024.12.0+467). Statistical analyses were supported by the following R packages: dplyr for data wrangling, ggplot2 for data visualization, car for Levene test of homogeneity of variance, rstatix for Welch ANOVA and Games-Howell post hoc tests, and lmtest for linear model diagnostics (eg, Breusch-Pagan test). These packages were used to perform descriptive statistics, regression models, nonparametric tests, and visualization of engagement metrics. The full analysis script is available via the OSF [84]. We analyzed the feasibility domains of acceptability, demand, implementation, practicality, integration, and expansion. Descriptive statistics and inferential methods were applied as appropriate. All significance tests were 2-tailed with an alpha level of *P*<.05 unless stated otherwise. Detailed analytic procedures for each domain are provided in Multimedia Appendix 5.

#### Qualitative Data Analysis

Qualitative data underwent content analysis based on the methods outlined by Mayring and Fenzl [77], utilizing MAXQDA (VERBI software). This content analysis focused on the dimensions of acceptability, demand, implementation, practicality, and limited efficacy testing. A deductive-inductive coding approach was used: A preliminary category system based on the framework by Bowen et al [50] guided the initial coding, and new categories were added inductively after reviewing 25% of the data. This approach ensured both theoretical grounding and responsiveness to participant insights.

The unit of analysis was the individual interview. Adolescent statements were analyzed at a moderate level of abstraction to maintain depth and thematic clarity. The selection criteria focused on statements from young people regarding their evaluation of the program and its feasibility.

The coding team consisted of the first author and 2 trained research assistants. Intercoder reliability reached *κ*=0.90 after iterative refinement, indicating a high level of agreement among coders [85,86]. Disagreements were resolved through discussion until consensus.

Reflexivity was emphasized throughout the coding process. Coders were trained to identify their own assumptions and ensure neutrality, particularly when interpreting subjective feedback [82]. The final category system included domains, subcategories, definitions, and anchor examples. It was continually adjusted to best represent the data (see Multimedia Appendix 6).

**Figure 2.**
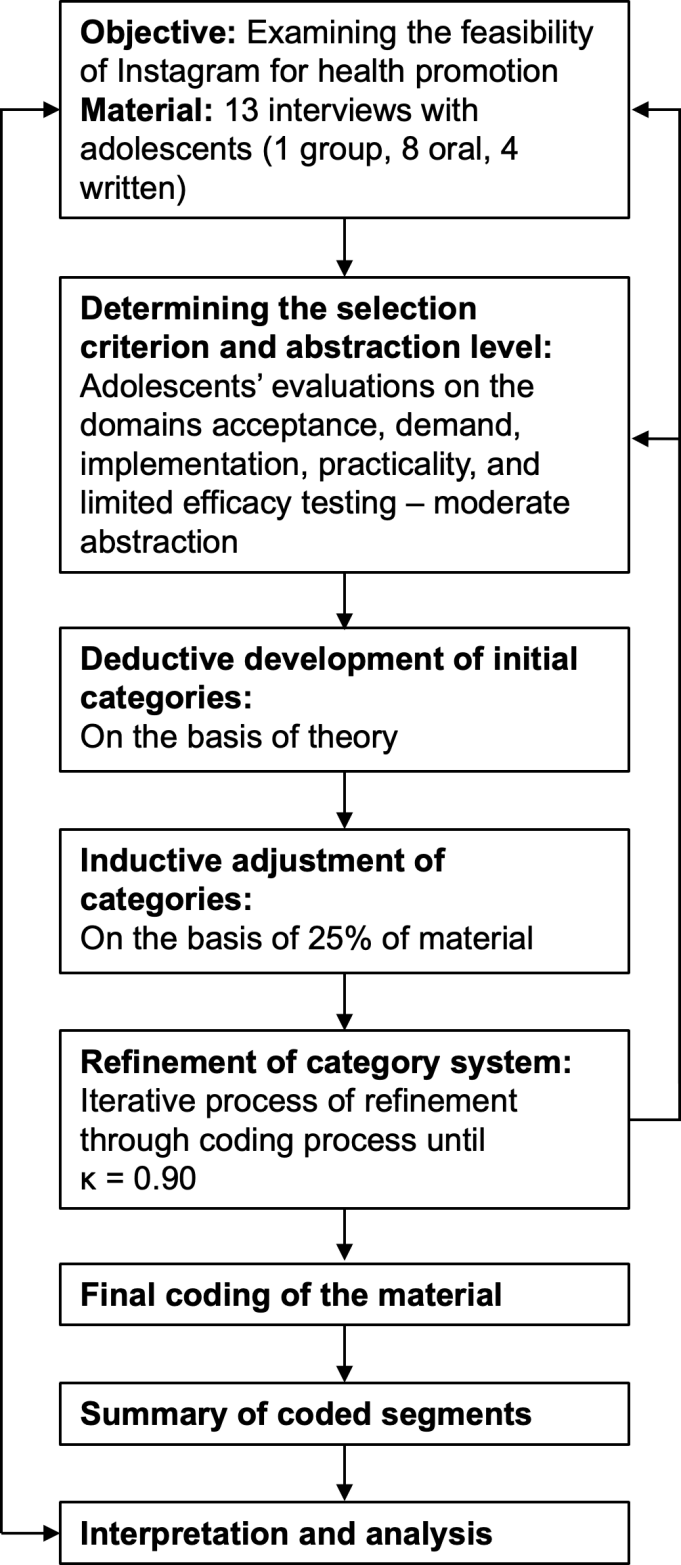
Qualitative content analysis process for adolescent preferences on the feasibility of the Instagram-facilitated life skills program leduin.

Finally, we mapped summarized results onto the original feasibility domains to confirm that our analysis addressed the study’s core research questions. Figure 2 summarizes the content analysis process.

## Results

### Overview

The following results draw on both quantitative and qualitative data to assess the feasibility of the leduin program. Quantitative findings are based on Instagram usage metrics and reflect behavioral engagement patterns across the entire sample (n=99). In contrast, qualitative findings stem from in-depth interviews with a purposively selected subsample (n=13), capturing participants’ subjective perceptions. Interview participants were selected based on their level of challenge participation—categorized as high (>5 participations), medium (1 to 5 participations), and low or none—to reflect varying degrees of engagement.

Within the qualitative results, high and medium challenge engagement was often associated with more differentiated feedback, greater motivation, and stronger signs of impact. However, consistently low or no engagement tended to co-occur with limited feedback and interest, reduced program consumption, and an overall weaker connection to the program. These patterns help contextualize the qualitative findings and offer a nuanced understanding of how adolescents experienced the program.

### Acceptability

#### Retention Rates

**Figure 3.**
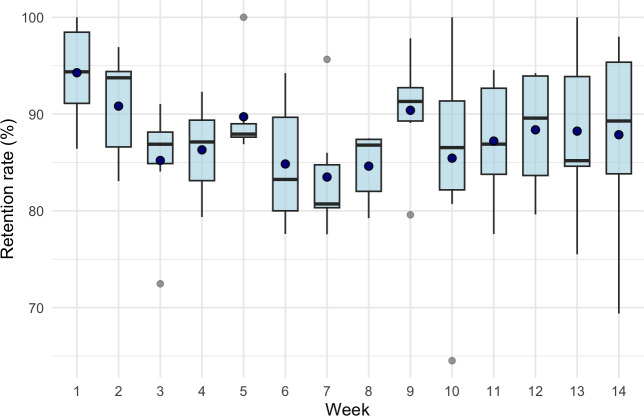
Retention rate distribution by week for the 14-week life skills program leduin.

Retention rates were calculated daily as the proportion of accounts that viewed the entire story, determined using the viewing of the final story frame. The rates ranged from 64.5% to 100% across the 14-week program, with views of each story ranging from a minimum of 34 to a maximum of 81. The average daily retention rate was 87.7% (SD 7.8%), indicating consistently high viewer engagement. Visual inspection suggested a slight dip in retention during the early weeks, followed by a stabilization in later weeks. Weekly boxplots confirmed stable medians with minor fluctuations and no substantial drop-offs, supporting the interpretation of sustained engagement throughout the intervention (see Figure 3).

#### Interaction Rates

**Figure 4.**
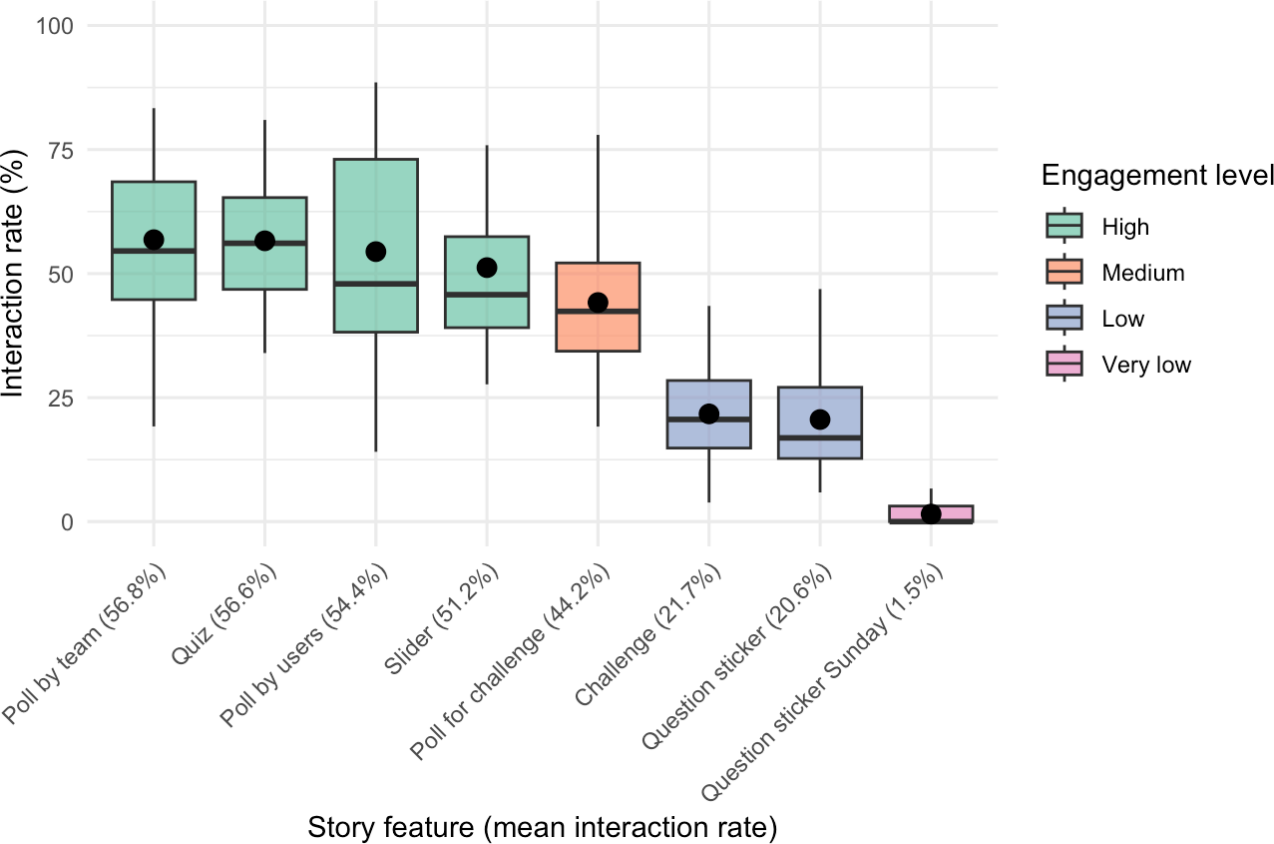
Interaction rates by story feature.

Interaction rates were determined as the proportion of accounts viewing the feature who actually interacted with it, and mean interaction rates were determined across all instances of each feature over the 14 weeks. Poll by team (mean 56.8%), quiz (mean 56.6%), poll by users (mean 54.4%), and slider (mean 51.2%) formed the group with the highest interaction rates, with no significant differences among them (poll by team vs quiz: *P*=.99; poll by team vs poll by users: *P*=.98; poll by team vs slider: *P*=.99; quiz vs poll by users: *P*=.97; quiz vs slider: *P*=.98; poll by users vs slider: *P*=.98). Poll for challenge (mean 44.2%) was significantly lower than these 4 (*P*<.001) but higher than challenge (mean 21.7%) and question sticker (mean 20.6%), which did not differ significantly from each other (challenge vs question sticker: *P*≥.99). Question sticker Sunday (mean 1.5%) had the lowest interaction rate, significantly lower than all other features (*P*<.001). These results demonstrate high content engagement through retention and substantial variability in interaction, depending on feature type, as visualized in Figure 4.

### Qualitative Acceptability Findings

#### General Evaluation of the Program

Participants perceived the program as highly valuable and relevant to their everyday challenges, including planning for the future, managing relationships, and regulating emotions. The promotion of life skills was seen as particularly important, with several participants noting a lack of such content in traditional education. The program was experienced as a safe space fostering self-reflection and personal growth. One participant summarized the following:

We live in times when young people struggle to find their way...I think this program is a great starting point....We don’t learn this in school....Maybe it will evolve or serve as a foundation for helping young people develop essential life skills....People my age, around 16, can find in this program a valuable retreat to grow and cope with their struggles.

[interview 3, section 103]

Many participants stated that they would recommend the program to their peers.

#### Evaluation of the Account Holder or Team

Participants described the program team as competent, trustworthy, and supportive. They reported feeling taken seriously, describing the team as inclusive and treating participants with equality and respect. At the same time, some adolescents expressed a desire for a more personalized and less research-influenced approach.

#### Language

Overall, adolescents found the program’s language appropriate and easy to understand. As one participant noted: “The language was well-suited – not overly sophisticated, but exactly at our level*”* (interview 06, sections 11‐12). This view reflected the majority opinion. However, 2 contrasting views emerged: One found the tone too formal, while another found it too simplistic and suggested more technical terms for better engagement.

#### Other Design Aspects

Participants praised the program’s well-structured and engaging design, which was free of unnecessary or dull elements. The playful and colorful layout—with vibrant graphics, illustrations, images, and practical tips—was seen as visually appealing and helpful for maintaining attention. One participant stated:

*The program was creatively designed, visually appealing, and colorful, which made reading enjoyable and helped maintain focus*.

[interview 04, section 35]

#### Interactivity and Anonymous Exchange

Interactive Instagram features such as question stickers, polls, and quizzes were highly appreciated, especially for enabling anonymous participation. In this context, anonymity not only meant expressing one’s own thoughts without revealing identity but also engaging in anonymous social comparison—seeing aggregated group responses without knowing who gave which answer. This allowed participants to compare their experiences with others, recognize commonalities, and feel less alone in their struggles. Many noted that anonymity enabled engagement, as discussing sensitive topics with strangers felt uncomfortable—an openness that they felt was impossible in a school context. One participant noted the following:

Being anonymous is great because it allows you to be more open and honest. You can complete the tasks based on what you truly think, without worrying that someone will recognize you. I also liked that questions were answered and that we received a message after each challenge. It was nice to see that people appreciated our participation.

[interview 01, sections 28-40]

Interaction with the program team was also valued. Participants appreciated receiving responses to their questions and personalized messages following challenge completion. Although the direct communication was initially surprising to some, it became a welcomed and appreciated feature.

Suggestions for improvement included expanding anonymous interaction options, such as incorporating anonymous video calls, and offering opportunities for one-on-one conversations with the team to discuss personal matters more deeply.

#### Instagram Features in Detail

Adolescents interacted with the program’s Instagram features in diverse ways, ranging from occasional to frequent use. The variety of available formats catered to different preferences and engagement styles. Low-threshold features like polls and quizzes were particularly well received for their ease of use, while more involved formats such as challenges or open-ended questions appealed to those seeking deeper engagement. Many participants valued the overall variety rather than preferring a single feature.

Despite this positive reception, several usability barriers emerged across features. Adolescents reported that cognitively demanding or time-consuming formats could lead to disengagement. Suggestions included simplifying language, improving visual clarity, and limiting content length.

#### Reels and Stories

The program’s reels and stories were regularly consumed, reflecting Instagram’s integration into adolescents’ daily routines. Reels were generally perceived as informative and concise; however, some lacked engaging visuals or felt cognitively demanding. Participants suggested making them shorter and more visually dynamic. Similarly, long or content-heavy stories were sometimes skipped, which disrupted comprehension. Some participants preferred static posts for their clearer structure and easier readability. Reducing the number of story slides per topic was a common suggestion to enhance accessibility.

#### Question Stickers, Quizzes, and Polls

The interactive features in the Instagram story were evaluated positively, although engagement levels varied. Polls stood out for their simplicity and one-click ease. Their immediacy and low demand made them a consistent tool for engagement. Quizzes were seen as fun and educational, with instant feedback enhancing motivation. However, mismatched answer options occasionally caused confusion. Question stickers encouraged self-expression and peer engagement but required significant effort. Although some used them actively, others ignored them due to the mental load of formulating answers.

#### Challenges

By those who participated in them, challenges were enthusiastically received. Many enjoyed the creative tasks like fill-in-the-template exercises, and small prizes boosted motivation. Still, others found multistep tasks too effortful. One participant noted: “It was exhausting to screenshot the stories and transfer them to my iPad because I couldn’t edit them on my phone” (interview 06, section 18). Others missed challenge posts or felt less motivated after winning once. Confusing instructions, especially when introductory content was missed, also posed barriers.

### Demand

**Figure 5.**
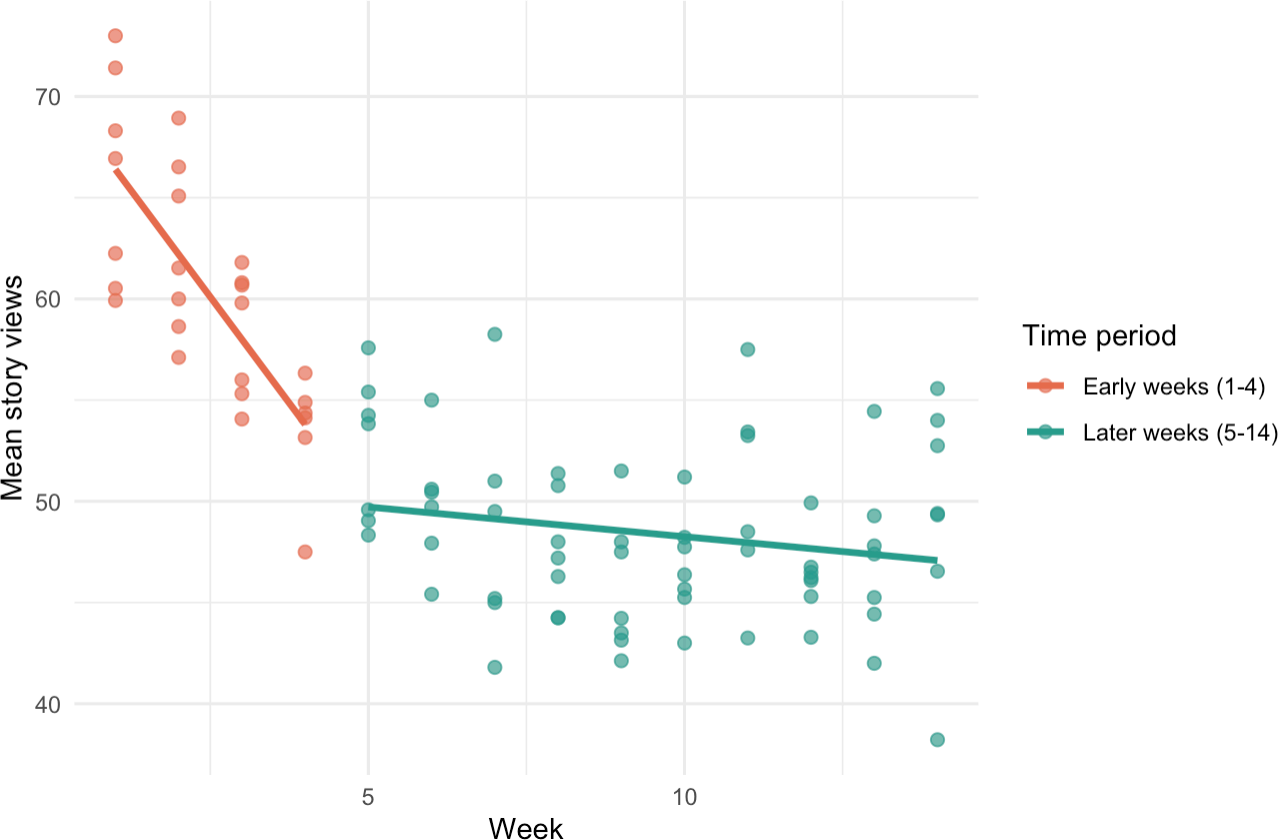
Comparison of story view decline between early weeks (1-4) and later weeks (5-14).

Story views showed a significant negative correlation with time (*r*=–0.70, *P*<.001), indicating a steady decline in engagement, with a maximum of 81 views and a minimum of 34 views. A linear regression analysis confirmed this trend (*ß*=–1.24, SE=0.13, *P*<.001, *R*²=0.49). However, residual diagnostics indicated nonlinearity, prompting a segmented regression analysis. The segmented model revealed a sharp decline during the first 4 weeks (*ß*=–4.20, SE=0.74, *P*<.001), followed by a nonsignificant slope thereafter (*ß*=–0.29, SE=0.18, *P*=.10). Figure 5 visualizes this segmented pattern clearly, showing the steep drop in story views during the early weeks and the relative stabilization of engagement thereafter. This model explained 69% of the variance and met the assumption of homoscedasticity (Breusch-Pagan test, *P*=.33). These results indicate that engagement declined primarily in the early phase and then stabilized, suggesting that demand was shaped more by timing than by specific content.

### Qualitative Demand Findings

#### Personal Relevance

Many adolescents reported a strong personal connection to the program content. Topics such as hate speech, social media use, cyberbullying, and substance use were perceived as especially relevant. Social media was frequently cited as an area where participants struggled and appreciated guidance. One participant reflected: “I found the content about social media the most helpful because so many young people, including myself, struggle with it. It was really valuable to receive education on this topic*”* (interview 08, section 2). Others noted how the content helped them better understand the blurry line between jokes and harmful behavior online.

Stress management was another core concern, with 10th grade described as a particularly stressful time. Adolescents also valued guidance on goal setting and future planning, often expressing uncertainty about their goals. Furthermore, interpersonal relationships, emotional regulation, and communication were considered highly relevant. As one participant put it: “The program focused a lot on friendships, social interactions, handling emotions, and conflict resolution, which I found really, really interesting*”* (interview 04, section 3). Self-care, self-reflection, and identity development were also frequently mentioned as particularly impactful. One participant shared: “The self-care challenge...really opened my eyes*”* (interview 05, section 4).

However, not all topics resonated equally. Some adolescents found content such as source evaluation or addiction awareness less relevant, particularly if these were already covered in other settings. Although some found all topics manageable, others described certain subjects such as hate speech or family conflicts as emotionally challenging or overwhelming.

#### Repetition

Opinions on the program’s variety were mixed among participants. Several participants appreciated the rotation of weekly topics and the structured, balanced presentation, describing the content as well-paced and thoughtfully sequenced. One participant commented: “I had the feeling that all the topics were somehow treated equally well and that it was well timed and built on each other*”* (interview 06, section 23).

Others felt that the program included too much repetition. The recurring Monday self-care challenge and continuous emphasis on behavioral change were seen by some as redundant or even demotivating. As one participant stated: “At some point, it gets boring to keep seeing or hearing the same thing — that you’re doing something wrong or that you need to improve*”* (interview 07, section 23).

### Implementation

Consistent with the demand analysis, story views declined sharply in the early weeks and stabilized thereafter. By week 4, 76% of the total drop in views had occurred, decreasing from a mean view of 66.1 in week 1 to 53.4 in week 4. By week 6, this figure rose to 97.3% (mean views 49.9). Although minor fluctuations were observed in later weeks, weekly averages remained stable, indicating that most disengagement happened early in the intervention.

### Qualitative Implementation Findings

Overall, adolescents reported that the program met their expectations. Most felt well-informed about its structure and content in advance, which helped avoid surprises during participation. They had anticipated a focus on social interactions, personal challenges, and mental health support, along with learning opportunities—areas they felt were well addressed. Some participants who were initially skeptical noted that the program exceeded their expectations.

### Practicality

Content was successfully published according to the predefined plan. Posts were scheduled around 2 PM to align with school dismissal times, ensuring availability throughout the afternoon and evening.

Mean views were distributed equally across weekdays (Kruskal-Wallis *χ*²_6_=0.92, *P*=.99). To examine whether content structure influenced retention, we explored associations between the number of story slides and viewer retention, as well as differences across weekdays. Story slides varied significantly during the program, ranging from 3 to 25 slides (mean 9.5, SD 4.9 slides). The Spearman rank correlation between the number of story slides and retention rate, however, was not statistically significant (*ρ*=0.11, *P*=.28), indicating no monotonic relationship between content length and viewer retention. Similarly, the correlation between weekday (Monday to Sunday) and retention rate was nonsignificant (*ρ*=–0.07, *P*=.50). These findings suggest that neither the amount of content per day nor the weekday of publication significantly influenced retention.

### Qualitative Practicality Findings

#### Total Duration and Daily Time Expenditure

Adolescents had mixed views on the program’s overall duration. Many found the 3-month period manageable and felt time passed quickly due to regular engagement and other commitments. One participant stated: “I found it very entertaining, it was very short. Yes, it was over quicker than expected*”* (interview 06, section 35). Others, however, felt it was too long, which led to decreased motivation and drop-off.

The daily time commitment, however, was generally seen as appropriate. Many noted that tasks could be completed in a few minutes and fit well into their Instagram routines. One participant explained: “The time required was fine since the program only took a few minutes per day” (interview 05, section 14). Still, some adolescents found it harder to keep up during busy periods due to academic demands.

#### General Frequency of Use

Engagement levels varied. Some participants followed the program closely, interacting several times per week or almost daily. One participant stated: *“*I saw most of it – I didn’t really miss anything” (interview 01, section 4). Others had moderate engagement, while a few interacted with only a small portion of the content and completed few or no tasks.

#### Pace of Topic Delivery

Feedback on pacing was also mixed. Some adolescents appreciated the structure and felt the time for each topic was sufficient. One participant noted: “I think the timing of the topics was appropriate. They weren’t covered too quickly, and there was enough time between them” (interview 01, section 16). Others found certain topics too long or overwhelming when presented with too much information at once.

#### Difficulty

Most participants found the content accessible and well-designed. Challenges were seen as doable, and videos were considered clear and easy to follow. However, tasks requiring deep reflection or decision-making in ambiguous situations were perceived as more difficult. One participant explained: “Too difficult? Well, there were challenges where I had to think more, but it was still appropriate, yes. So it was still doable.” (interview 02, section 42).

#### Integration

To recruit adolescents for the leduin program, we contacted schools in North Rhine-Westphalia and youth centers across Germany. In total, we contacted 101 schools: 52 grammar schools, 35 secondary schools, and 14 free schools. We contacted 10 youth centers, and we held 31 information events in 19 schools and 1 youth center (reflecting 20, or 18.2%, of the initially contacted 111 institutions) with approximately 775 students, with an average of 25 students per class.

Of the 775 students reached through the information events, 99 decided to participate in the study, 10 (10%) coming from 1 youth center and 89 (90%) from 6 different schools (4 secondary schools and 2 grammar schools), resulting in an overall participation rate of 12.8% (99/775). At Instagram, a maximum of 98 followers was noted, showing that nearly all participants who agreed to participate in the study and the program also followed the account. These results indicate that initially reaching the target group was hard despite high interest from teachers and social workers.

### Limited Efficacy Testing

#### Subjective Learning Success

Many adolescents reported meaningful personal growth from participating in the program. They described increased self-confidence, enhanced stress management, and a more constructive approach to everyday challenges. Participants noted greater self-awareness, authenticity, and attentiveness to their own needs, often applying strategies such as writing to-do lists to stay organized. The program also encouraged reflection, normalized mistakes, and supported the development of clearer future goals and a more optimistic outlook.

Social benefits were equally prominent. Adolescents described improved communication and emotional expression, greater empathy, and stronger conflict resolution skills. Many felt more comfortable forming new friendships and more capable in social situations. The program also influenced how participants engaged with social media. Increased awareness of cyberbullying, hate speech, social comparison, and digital well-being led to behavioral change, including unfollowing negative accounts, limiting screen time, reporting harmful content, and focusing on inspirational profiles. One participant summed up their experience:

I’m now more mindful of my Instagram use. I set time limits — sometimes 10 minutes, sometimes just 5. I only follow people who inspire and uplift me, which has helped my mental health. In my daily life, I apply new techniques. When I feel stressed, I create a to-do list and work through it. I’ve also started thinking about my future and setting goals. I try to stay positive, even in conflicts. I see mistakes as learning opportunities and try to grow from them. My communication has improved, both in real life and on Instagram. I can express my feelings better, which has strengthened my relationships. What I’ve taken away from the leduin program is that making mistakes is okay and that you should always keep your goals in mind.

[interview 07, sections 40-55]

#### Limitations and Lack of Perceived Impact

Although most participants reported positive outcomes, some felt the program had limited relevance or impact. Some attributed this to prior knowledge, noting they learned little that was new. One person reported no significant change in their behavior or social interactions, stating the following:

I didn’t notice any changes in my behavior on Instagram that could be directly linked to the program. Maybe it’s because I didn’t follow along consistently. Unfortunately, I also didn’t find any real-life applications for what was taught — neither in how I treat myself nor in how I interact with others. There’s nothing I personally took away from the leduin program or benefited from.

[interview 03, sections 66-77]

### Adaptation

Adaptation considers the extent to which an intervention can be modified to suit different contexts or audiences. In this study, the domain was not empirically assessed but considered conceptionally, as the program was intentionally designed as a fixed 14-week intervention. This structure was grounded in established theory and prior evidence from behavioral change research and life skills education and developed using a co-design approach to ensure alignment with adolescents’ needs and preferences. Although the program was not designed for real-time modification, it demonstrated responsiveness through interactive features and anonymous exchange, including reposting of participant input. These elements created space for individualized engagement, even though the content itself remained unchanged. Accordingly, the program’s limited flexible adaptation options were a conscious decision to maintain its theoretical integrity and consistency.

In addition to conceptual scalability, we estimated the staff resources required for recruitment and delivery. The calculation included initial and follow-up contact with 111 institutions (15 minutes each; ≈27.75 hours); 31 online information events including preparation (1 hour each; ≈31 hours), coordination, and consent form processing for 99 participants (10 minutes each; ≈16.5 hours); daily content posting and moderation during the 14-week program (1 hour/day; 98 hours); and weekly newsletters to institutions (15 minutes/wk; ≈3.5 hours). In total, these activities accounted for ≈155 hours, corresponding to an average of about 94 minutes (1 hour, 34 minutes) of staff time per participant, excluding evaluation activities.

## Discussion

### Study Overview

In this pilot study, we used a mixed methods approach to evaluate the feasibility of leduin, a 14-week Instagram-based prevention program designed to promote life skills and functional social media use among adolescents. A total of 99 participants aged 14 years to 18 years were recruited through schools and youth centers in Germany. The program delivered short, interactive daily content via a private Instagram account. Feasibility was assessed through an adapted version of the framework by Bowen et al [50], combining quantitative platform usage metrics with qualitative in-depth postprogram interviews to capture adolescents’ experiences and perspectives.

### Integration of Feasibility Domains

Taken together, the leduin program demonstrated high feasibility, with its theory-based and co-designed format aligning closely with adolescents’ preferences and daily habits. Findings indicate high acceptability, supported by a mean retention rate of 87.7% across the 14-week program, surpassing industry benchmarks of approximately 73% retention by the 10th frame, which corresponded to the average story length in this study [87]. Retention serves as a strong indicator of acceptance, especially given that Instagram stories function independently, do not compete with other content, and, once started, remain unaffected by algorithmic influence. These rates remained stable regardless of the number of story slides (ranging from 3 to 25) or the weekday of posting and did not follow the expected linear decline. In some cases, view counts even increased toward later story frames, suggesting that adolescents engaged selectively with content rather than dropping off systematically. This highlights the importance of interpreting retention within context rather than presuming a continuous decrease.

High engagement was further reflected in interaction rates, which revealed a clear hierarchy based on the effort required. Low-effort features showed the highest interaction: team polls (56.8%), quizzes (56.6%), user-generated polls (54.4%), and sliders (51.2%), with adolescents favoring brief, relatable formats that enabled peer comparison. The lack of significant difference between team- and user-generated polls suggests that topic relevance outweighed the importance of source. Polls linked to challenge prizes (44.2%) showed lower engagement, likely due to reduced emotional or social resonance. Interaction dropped further for more cognitively and technically demanding features such as challenges (21.7%) and question stickers (20.6%). The open-ended Sunday questions recorded the lowest interaction rate (1.5%), likely due to limited perceived relevance.

Qualitative data further supported the high acceptability of the program’s language, design, and structure. Participants described the content as visually appealing, accessible, and thoughtfully presented. Interactive elements, particularly those allowing anonymous engagement, were valued for fostering openness and inclusion. These findings align with prior research highlighting the effectiveness of relatable visuals, authentic language, culturally resonant messaging, and interactive features in social media-based health interventions [45-48].

Moreover, adolescents considered the daily time requirement manageable, the pacing appropriate, and most tasks feasible, even though perceived difficulty varied individually. This indicates that the program integrated well into daily routines. Its flexible and low-effort design aligns with research emphasizing the unique advantages of social media as a platform for youth health promotion such as cost-effectiveness, accessibility, and seamless integration into everyday behavior [3,21-25]. In sum, the program’s methods and structure enabled interactivity and individualized engagement without requiring real-time content adjustments, thereby maintaining theoretical integrity and consistency.

In doing so, the program addressed key developmental tasks and interests. After an initial drop in story views during the first 4 weeks, interest stabilized without topic-specific spikes, indicating sustained and evenly distributed demand across all content. This pattern likely reflects a common phenomenon in digital health interventions: Adolescents may begin with a willingness to participate but disengage when content does not feel immediately relevant [88,89]. Content interaction is further influenced by Instagram’s algorithm: However, as academic evaluations of Instagram’s algorithmic principles are scarce, such insights rely largely on industry analyses [90]. These indicate that content visibility is strongly influenced by the platform’s algorithm, which prioritizes posts and stories from accounts with which users interact most frequently. Consequently, low interaction early in the program may have triggered reduced algorithmic ranking, meaning fewer followers were shown subsequent posts in their feeds or story lists. This is because Instagram’s algorithm weighs both an individual’s past engagement with the account and the overall interaction rates across the audience, so lower aggregate engagement can diminish visibility even for followers who have not actively disengaged. This creates a feedback loop in which reduced exposure further limits collective and individual opportunities for re-engagement, potentially accelerating dropout. Although this dynamic challenges scalability, the stabilization of views in later weeks suggests that participants who found the content meaningful continued engaging despite algorithmic filtering. That supports the feasibility of sustained delivery for an interested core audience.

Qualitative data corroborated this interpretation, as most adolescents described the topics as relevant to their daily lives. However, preferences for specific content areas and views on content repetition varied individually. Functional social media use, cyberbullying, and digital violence were frequently cited as particularly meaningful. These concerns align with existing evidence on adolescents’ vulnerability to problematic social media use, including exposure to harmful content, cyberbullying, and addictive platform mechanisms [13-16]. These risks correlate with mental health issues such as depression, anxiety, and stress [17-19], reinforcing the need for interventions that promote emotional resilience and digital literacy [20]. Accordingly, many participants reported increased awareness of their media use and a growing desire to adopt more intentional digital habits.

In line with the WHO life skills framework [67] and the Ottawa Charter for Health Promotion [66], participants also identified interpersonal relationships, emotional regulation, and school-related stress as central concerns. This alignment indicates the program’s developmental relevance. Delivered in 10th grade—a transitional phase marked by academic pressure, peer dynamics, and important life decisions—the program was well timed, consistent with findings showing that life skills interventions are particularly effective during this stage [70,91].

Despite high engagement and perceived relevance during the program, recruiting adolescents initially proved challenging. Outreach through schools and youth centers resulted in a participation rate of only 12.8% among the 775 students directly approached through information events, with the research context likely serving as an additional barrier. Unlike traditional school-based programs that reach adolescents within the classroom [92], leduin relied on voluntary participation during adolescents’ free time. Although this approach limited the sample size, it generated a focused but self-selected group. Nearly all who enrolled followed the Instagram account, indicating alignment between expressed interest and actual behavior. Qualitative data confirmed this: Most participants stated that the program met or exceeded their expectations, which likely contributed to continued engagement. However, the recruitment challenge remains. Digital outreach, although an intuitive alternative, does not ensure better access. For example, even a well-targeted Instagram campaign failed to reach their intended audience, with less than 7% of followers ultimately belonging to the target group [47]. Engaging adolescents in voluntary prevention programs thus remains a key concern for future efforts. At the same time, despite the challenges in recruitment, the resources invested per participant—about 94 minutes for recruitment, program implementation, and organizational support—can be considered economical given that each adolescent received a 14-week intervention with daily input and first indications of positive impact.

Overall, the findings suggest that a structured, Instagram-based life skills program offers a feasible and promising approach to adolescent health promotion. Its modular structure, developmental fit, and flexible delivery model provide a strong foundation for expansion to other target groups or more specific health-related topics.

### Strengths and Limitations

This study offers several notable strengths that enhance its methodological integrity and relevance for advancing research on digital prevention. First, the mixed methods design, based on the feasibility framework by Bowen et al [50], enabled a comprehensive evaluation by combining behavioral Instagram engagement data with adolescents’ subjective perceptions of their motivations, the program’s perceived impact, and usability.

Although usage data reflect direct engagement with the content and program methods, the semistructured interviews allowed for an in-depth, participant-driven exploration of program perceptions, barriers, and outcomes [80]. The deductive-inductive coding strategy ensured that analysis was grounded in theory while remaining open to emergent insights. High intercoder reliability (κ=0.90) confirms the consistency of the qualitative analysis [86], and the reflexive approach adopted by the research team helped to minimize bias during interpretation [82].

However, the study also presents limitations that restrict the generalizability and interpretability of the findings. Most notably, the sample was limited to adolescents in Germany, largely drawn from schools and youth centers in specific regions, which limits external validity. This is because health perceptions and social media use are influenced by cultural and regional differences regarding, for example, usage patterns, privacy concerns, or self-disclosure habits [93-96]. Voluntary participation may have led to self-selection bias, overrepresenting adolescents with higher motivation or interest in psychological topics [97]. Although valuable for understanding feasibility, this limits insights into broader population-level uptake and effectiveness.

To address this, it is useful to consider cross-cultural perspectives on digital literacy and social media use. A recent overview synthesizes global evidence, showing that, in many majority-world countries, social media plays an even greater role in young people’s daily lives than the internet in general [98]. At the same time, access is less universal than in Western countries, digital skills are often lower, and social media is more heavily relied upon for information seeking. The overview also highlights gender inequalities, fewer opportunities for parental or school-based digital literacy support, and less regulation for youth. Taken together, these conditions suggest that using social media to impart skills that protect and empower young people could be even more critical in these contexts than in settings like Germany.

Further, the integration of the intervention into school settings also proved difficult, with a low participation rate of 12.8% despite strong institutional support. This challenge in reaching adolescents outside of class time highlights a key barrier to scalability and suggests that more targeted recruitment strategies are needed in future studies as well as further insights into what factors support program participation.

Additionally, social desirability bias may have influenced interview responses, despite the team’s efforts to create a safe, trusting environment. The dual role of the researchers as program implementers and interviewers could have subtly shaped how adolescents expressed their views [99], even though the main account holder did not conduct interviews to reduce this risk. Furthermore, member checking was not performed, meaning that participants did not have the opportunity to review or confirm the researchers’ interpretations of their interviews, potentially affecting the accuracy of qualitative findings [100].

Finally, although the study demonstrates promising results in terms of perceived impact and engagement, no long-term follow-up was conducted, and effectiveness was only evaluated through subjective self-report. This limits conclusions about sustained behavioral change or broader health outcomes. Although we deliberately chose to use a private Instagram account to ensure confidentiality, trust, and privacy, incorporating direct Instagram analytics in future studies would be valuable. Such data, alongside smartphone-based passive sensing combined with self-report, could enrich feasibility evaluations by providing objective verification of engagement patterns, contextualizing self-reported experiences, and quantifying relationships between online activity and well-being. Although these approaches come with ethical and practical challenges, particularly regarding privacy and compliance, they hold considerable potential for capturing adolescents’ behaviors more comprehensively and for linking subjective perceptions with observable usage data [101,102].

### Implications for Future Research, Policy, and Practice

Despite its limitations, this study provides robust preliminary evidence on the feasibility of using Instagram to promote life skills among adolescents and offers important methodological guidance for digital prevention research. Future research should focus on strategies to improve initial recruitment and reduce early dropout, both of which pose key challenges in voluntary, free-time programs. To address self-selection bias and enhance generalizability, future studies should include more diverse and geographically broader samples. Beyond subjective reports, controlled studies are needed to assess the program’s efficacy and long-term impact. Research should also explore algorithm-informed content strategies to optimize visibility and engagement, as well as the expansion potential of Instagram-based interventions for other age groups and health topics.

In practice, this study provides actionable insights for designing engaging social media interventions. Usage metrics revealed clear preferences: Low-effort features (eg, polls, sliders) encourage routine interaction, while more cognitively demanding features (eg, challenges, open questions) are best used strategically when deeper reflection is desired. This underscores the importance of feature sensitivity and intentional design. The sustained engagement observed supports the feasibility of delivering even complex interventions via Instagram when content is relevant and well-integrated into adolescents’ daily routines. Co-design was essential for aligning with adolescents’ preferences, reinforcing the importance of participatory development. Notably, the high demand for emotionally and socially relevant topics confirms the need for prevention programs that directly address the realities of adolescents’ lives.

Policy should acknowledge social media platforms as viable channels for health promotion. This includes supporting the development and evaluation of evidence-based, culturally sensitive digital interventions and providing funding frameworks that support innovative formats to go beyond traditional school-based models. National prevention strategies should explicitly include digital interventions as low-threshold, scalable complements to in-person services particularly for reaching youth where they already are: on social media.

### Conclusions

This pilot study demonstrates that delivering a structured, theory-based life skills intervention via Instagram is both feasible and promising. High engagement rates, positive user feedback, and meaningful self-reported outcomes suggest that social media can serve as a powerful platform for adolescent health promotion, especially when interventions are co-designed, culturally aligned, and intentionally integrated into daily routines. Although initial recruitment and early disengagement remain key challenges, the overall findings underscore Instagram’s potential for complex, sustained interventions. Building on this foundation, future research and policy should continue exploring how to effectively harness digital environments to support adolescent development and mental well-being.

## Supplementary material

10.2196/78774 Multimedia Appendix 1 Description of the development of the leduin program.

10.2196/78774 Multimedia Appendix 2 Adaptation of the feasibility framework by Bowen et al [50] for this study.

10.2196/78774 Multimedia Appendix 3 Overview of interview topics and their corresponding feasibility domains.

10.2196/78774 Multimedia Appendix 4 Interview guide for semistructured interviews with participants of the leduin program.

10.2196/78774 Multimedia Appendix 5 Detailed analytic procedures for each domain.

10.2196/78774 Multimedia Appendix 6 Category system for qualitative content analysis of semistructured interviews with adolescents.

## Abbreviations

COREQ: Consolidated Criteria for Reporting Qualitative Research
JARS-Qual: Journal Article Reporting Standards for Qualitative Research
JARS-Quan: Journal Article Reporting Standards for Quantitative Research
MMARS: Mixed Methods Article Reporting Standards
OSF: Open Science Framework
WHO: World Health Organization
