# Supplementary material for: Feasibility of the Social Media–Based Prevention Program “Leduin” for German Adolescents on Instagram: Mixed Methods Pilot Study
Source: JMIR Form Res. 2025 Nov 27;9:e78774. doi: 10.2196/78774 (PMC12661607; doi:10.2196/78774)
Supplement: Multimedia Appendix 1 [file formative-v9-e78774-s001.docx]

**Appendix 1 - Description of the development of the leduin program**

The intervention was designed to promote adolescents’ digital life skills by translating the WHO life skills framework (WHO, 1999) into engaging, platform-specific formats suitable for Instagram.

Key psychological mechanisms targeted in the program include (a) personal variables such as attitudes, self-regulation, and self-efficacy based on social cognitive theory and the theory of planned behavior (Ajzen, 1991; Bandura, 2005; Fishbein & Ajzen, 2010), (b) environmental factors including social norms and support (Bandura, 1977; Bandura et al., 1961), (c) information processing mechanisms to optimize message impact (Petty et al., 1997; Petty & Cacioppo, 1986; Petty & Wegener, 1998), and (d) the activation of resources to foster digital life skills development (Grawe, 1998).

The overall logic model posits that strengthening these areas will improve life skills and functional social media use and through that support mental health and well-being, enhance functional social media use, improve school performance, and reduce engagement in risky behaviors. The program targets adolescents in 9th and 10th grades, typically aged 14 to 16, representing a critical developmental stage during which life skills education has been shown to be particularly effective. This transitional period often involves students either completing school or preparing to enter upper secondary education, making it a key window for intervention (Pedditzi et al., 2023; WHO, 1994). Additionally, adolescents aged 15 to 16 spend nearly twice as much time online as those aged 9 to 11, underscoring the relevance of addressing digital competencies in this age group (Smahel et al., 2020).

Consequently, a methodological framework specifically tailored to Instagram’s unique affordances was developed. This framework was informed by the behavior change technique taxonomy (Michie et al., 2013) and further integrated insights from research on social media-based behavior change (Elaheebocus et al., 2018; Simeon et al., 2020). Additionally, a co-design approach was employed, incorporating qualitative input from focus groups and interviews with 67 adolescents aged 14 to 17 from diverse educational backgrounds to ensure the program's relevance and appeal (Zimmermann & Tomczyk, 2025). Adolescents’ perspectives helped identify preferred design principles and interactive Instagram features. Adolescents emphasized visual appeal, interactivity, and relevance, while discouraging features perceived as intrusive or school-like. Preferences included short, subtitled reels, polls, quizzes, and anonymous participation options. They favored real, relatable people in the content and requested meaningful incentives like praise or raffles. These findings directly informed the program’s feature selection and delivery strategy.
