# Supplementary material for: Feasibility of the Social Media–Based Prevention Program “Leduin” for German Adolescents on Instagram: Mixed Methods Pilot Study
Source: JMIR Form Res. 2025 Nov 27;9:e78774. doi: 10.2196/78774 (PMC12661607; doi:10.2196/78774)
Supplement: Multimedia Appendix 2 [file formative-v9-e78774-s002.docx]

**Appendix 2 - Conceptualization of Bowen’s feasibility framework for leduin pilot study**

Acceptability refers to how participants perceive and respond to the intervention. In the leduin program, this was assessed through participant feedback on the usability of the program and interaction with its various Instagram features, as well as through quantitative engagement metrics.

Demand relates to the anticipated and actual interest and participation of the target group. We measured this both qualitatively through perceived relevance and interest, and quantitatively via usage metrics over time and across different topics.

Implementation traditionally refers to the degree to which an intervention is delivered as intended. In a digital setting, this was reinterpreted to focus on whether the program delivery met adolescents’ expectations and at which points engagement dropped.

Practicality assesses how feasible it is to integrate the intervention into users’ daily lives. Instead of provider-side feasibility, this domain was reoriented to focus on participants’ ability to engage with the content consistently and their perceived time and resource investment. Quantitative analyses looked at engagement patterns by weekday and content volume.

Adaptation considers the extent to which the intervention can be modified to suit different contexts or audiences. In this study, this domain was not empirically assessed due to the intervention’s intentionally fixed design, grounded in theory and supported by prior evidence. While the program was responsive in some ways – such as through reposting participant input – its structure and core methods were not designed to change in real time. Accordingly, adaptation was considered conceptually, acknowledging limited flexibility as a deliberate design choice to preserve theoretical integrity.

Integration refers to how well the intervention fits into existing systems or cultural practices. Since leduin operated independently of schools, we examined how schools can support its implementation. This reconceptualization acknowledges the school system's role in enabling rather than delivering the program.

Limited efficacy testing aims to capture preliminary indications of program outcomes. In our study, this was approached by asking participants to reflect on perceived changes in their digital behavior, interpersonal interactions, or stress management as a result of participating in the leduin program.

Expansion evaluates whether the intervention can be scaled to other populations or settings. We addressed the conceptional relevance of this domain and added an empirical evaluation of the resources spent to reach participants.

By evaluating these domains we aimed to assess the feasibility of delivering complex psychological interventions through social media and to inform the iterative refinement of the leduin program. An overview of the domains, their interpretation, and operationalization in this study is provided in table 1.
