# Supplementary material for: Feasibility of the Social Media–Based Prevention Program “Leduin” for German Adolescents on Instagram: Mixed Methods Pilot Study
Source: JMIR Form Res. 2025 Nov 27;9:e78774. doi: 10.2196/78774 (PMC12661607; doi:10.2196/78774)
Supplement: Multimedia Appendix 3 [file formative-v9-e78774-s003.docx]

**Appendix 3 - Overview of interview topics and their corresponding feasibility domains**

| Domain/category | Dimensions | Definition | Questions in the interview guide |
| --- | --- | --- | --- |
| (1) Acceptability | Valence | General perception of the program as rather good or bad. | How would you convince a friend to participate in the leduin program? |
|  | Evaluation of the account holder/the team | Assessment of the competence, trustworthiness and support provided by those responsible for the prevention program | Did you feel taken seriously within the leduin program? |
|  | Interactivity and anonymous exchange | Evaluation of the features and functions of the program that enable the active participation of young people, including the (anonymous) exchange with each other and with the program managers | How did you like being able to exchange anonymously within the program?  Would you have liked more or fewer opportunities for interaction? |
|  | Language | the way in which the linguistic design of the content of the prevention program is perceived. includes the formality, appropriateness and comprehensibility of the language (simple, appropriate, difficult) | Was the language appropriate? |
|  | Other design aspects | Evaluation of formal elements such as the user interface and navigation, layout, design, methods and organization | Inductively derived from statements made by the interviewees |
|  | Features | General evaluation, reasons and barriers of use and frequency of use for various features (Challenges, question stickers, quizzes, stories, polls, reels, | What prevented or motivated you to participate in the interactive features (surveys, quizzes, challenges)?  Which features did you like best (videos, surveys, anonymous exchange, quizzes, challenges)?  From your perspective, are there any boring, superfluous, or ineffective elements/features in the program? |
| (2) Demand | Personal relevance | Personal connection and individual relevance of the content (extent to which the program content matches personal experiences and interests) | Did you find the content interesting and enjoyable?  Which content did you find the most helpful?  Which topics in the leduin program did you find most interesting?  Is there something you would have liked to discuss anonymously in the program?  Did any of the content cause you distress? |
|  | Repetition | Evaluation of the diversity of different topics and perspectives within the program | Do you think we covered some topics too often or not enough? |
| (3) Implementation | Expectations | What participants expected from the program after the information events and if this was met. | What were your expectations for the leduin program, and were they met? |
| (4) Practicality | Total duration of the program | Evaluation of the time span over which the prevention program is implemented | How did you feel about the length of the program? |
|  | Daily time expenditure | Evaluation of the daily time required to use the program effectively and integrate it into everyday life | How did you find the time commitment associated with the program? |
|  | General frequency of use | How often the program and its content was consumed in general. | How often would you say you viewed the program's content?  How much did you participate in the interactive elements (surveys, quizzes, challenges)?  Did you participate in the challenges? |
|  | Pace of topic delivery | Evaluation of the tempo, how fast or slow the various topics are presented or treated in the program | Inductively derived from statements made by the interviewees |
|  | Difficulty | Extent to which participants find the program content too difficult, too easy or appropriate | Were you able to understand the content well?  Were the tasks (e.g., challenges) too difficult for you? |
| (7) Limited efficacy testing | Subjective learning success | Individual perception of changes in a person's thoughts and actions after participating in the program | Have you noticed any changes in your behavior on Instagram, and if so, what are they?  Have you noticed in daily life that you apply newly learned content? What are these?  Have you noticed changes in how you treat yourself?  Have you noticed changes in how you interact with others, both in real life and on Instagram?  What have you taken away from the leduin program for yourself? What have you benefited from the most? |
