# Supplementary material for: Feasibility of the Social Media–Based Prevention Program “Leduin” for German Adolescents on Instagram: Mixed Methods Pilot Study
Source: JMIR Form Res. 2025 Nov 27;9:e78774. doi: 10.2196/78774 (PMC12661607; doi:10.2196/78774)
Supplement: Multimedia Appendix 4 [file formative-v9-e78774-s004.pdf]

Interviews Jugendliche

Hinweise für die Interviewdurchführung:

Bitte halte Dich an den Interviewleitfaden soweit möglich. Es handelt sich um ein teilstrukturiertes Interview, auch Leitfadeninterview. D.h., dass die Fragen des Leitfadens alle gestellt werden sollen, Sprünge innerhalb der Fragen allerdings (wenn sie inhaltlich passen) und auch Nachfragen oder Erläuterungen erlaubt sind. Hier sind zwei mögliche Arten, konkreter nachzufragen oder aber auf das Thema zurückzukommen. Auf diese Weise kannst Du darauf dringen, dass möglichst alle Fragen auch beantwortet werden. Aber auch knappe Antworten der Jugendlichen sind völlig ok.

- *Der Aspekt XY, von dem du gerade gesprochen hast, ist sehr interessant. Magst du mir noch etwas mehr darüber erzählen?*
- *Dieser Aspekt ist sehr interessant, führt jedoch von meiner eigentlichen Fragestellung weg. Darf ich dich noch einmal fragen, was du mit XY meinst?*

Begrüßung

Hallo! Vielen Dank, dass Du Dich zu diesem Interview bereiterklärt hast und Dir Zeit dafür nimmst. Das wissen wir sehr zu schätzen. Ich freue mich, dass ich mich mit Dir über das leduin-Programm austauschen kann.

Du hast ja in den letzten Wochen und Monaten am leduin-Programm teilgenommen. Wir interessieren uns jetzt dafür, was Du dabei für Erfahrungen gemacht hast. Wir wollen unbedingt auch die Perspektive der Teilnehmenden berücksichtigen, wenn wir das Programm jetzt überarbeiten. Wir freuen uns auf Dein Feedback. Zögere nicht, auch kritisch zu sein. Das hilft uns sehr weiter.

Um Deine Aussagen später wissenschaftlich auswerten zu können, nehme ich unser Interview auf. Deine Aussagen bleiben aber immer anonym und können nicht Dir als Person zugeordnet werden. Die Einwilligung dazu hast Du ja bereits mit Deinen Eltern gemeinsam unterschrieben.

- - - Aufnahme starten - - -

Evaluation Programm

- Was würdest Du sagen, wie viel bzw. oft hast Du die Inhalte vom Programm angesehen?

- Wie viel hast Du an den interaktiven Elementen (Umfragen, Quizze, Challenges) teilgenommen und warum?
    - Was hat Dich davon abgehalten, teilzunehmen?
- Welche Inhalte fandest Du am hilfreichsten?
- Was hat Dir gut gefallen?
    - Fandest Du die Inhalte interessant und haben sie Spaß gemacht?
    - Konntest Du die Inhalte gut verstehen?
    - War die Sprache passend?
    - Welche Features haben Dir am besten gefallen (Videos, Umfragen, anonymer Austausch, Quizze, Selbstfürsorge, Stressbewältigung, Challenges, Workbook)?
    - Wie fandest Du den zeitlichen Aufwand, der mit dem Programm verbunden war?
- Was hat Dir nicht gut gefallen?
    - Gibt es aus deiner Sicht langweilige, überflüssige, wirkungslose Elemente/Features im Programm?
    - Haben wir aus Deiner Sicht Themen zu oft oder zu wenig behandelt?
    - Waren Aufgaben (z.B. Challenges) zu schwer für Dich?
    - Haben Dich manche Inhalte belastet?
- Was würdest Du anders machen bzw. sollten wir anders machen?
- Wie hast Du die Dauer des Programms empfunden?
- Hast Du an den Challenges teilgenommen? Warum (nicht)?
- Hattest Du das Gefühl, innerhalb des leduin-Programms ernstgenommen zu werden?
- Wie hat es Dir gefallen, dass Du Dich innerhalb des Programms anonym austauschen konntest?
    - Hättest Du Dir mehr Möglichkeiten zum Austausch gewünscht? Oder weniger?

Evaluation Implementierung

- Hast du Veränderungen in deinem Verhalten auf Instagram feststellen können und wenn ja welche?
- Ist dir im Alltag aufgefallen, dass du neu gelernte Inhalte anwendest? Welche waren das?
- Hast du Veränderungen im Umgang mit dir selbst feststellen können?
- Hast du Veränderungen im Umgang mit deinen Mitmenschen feststellen können, in der Realität aber auch auf Instagram?
- Was hast du aus dem leduin-Programm für dich mitnehmen können? / Wovon hast du am meisten profitiert?

Evaluation Rekrutierung

- Was hat Dich motiviert, an dem Programm teilzunehmen?
- Was hat Dich motiviert, dran zu bleiben und nicht im Verlauf des Programms irgendwann einfach aufzuhören?
- Wie würdest Du eine Freundin/einen Freund davon überzeugen, am leduin-Programm teilzunehmen?
- Was denkst Du, warum haben sich andere gegen die Teilnahme am Programm entschieden?
- Welche Erwartungen hattest du an das leduin-Programm und wurden diese erfüllt?

Exploration aktueller Themen

- Was sind aktuell die Themen in Deinem Leben, die Dich besonders beschäftigen?
- Wofür suchst Du aktuell nach Lösungen in Deinem Leben?
- Welche Themen im leduin-Programm fandest Du am interessantesten?
- Gibt es etwas, worüber Du Dich im Programm gerne anonym ausgetauscht hättest?

Wir haben nun alle Fragen besprochen, die wir vorbereitet haben. Hast Du noch Anregungen? Gibt es Themen, die wir nicht angesprochen haben?

Abschluss

- Hast Du noch Fragen?

Okay. Dann danke ich Dir ganz herzlich für das Interview. Du hast uns damit sehr geholfen und trägst dazu bei, dass wir unser Angebot noch besser für Jugendliche gestalten können.
