# Supplementary material for: Feasibility of the Social Media–Based Prevention Program “Leduin” for German Adolescents on Instagram: Mixed Methods Pilot Study
Source: JMIR Form Res. 2025 Nov 27;9:e78774. doi: 10.2196/78774 (PMC12661607; doi:10.2196/78774)
Supplement: Multimedia Appendix 5 [file formative-v9-e78774-s005.docx]

**Appendix 5 - Detailed analytic procedures for each domain**

**Acceptability**
We assessed acceptability by analyzing daily retention rates, defined as the proportion of viewers who watched an Instagram story to the end. We computed descriptive statistics (mean, median, SD, range) and applied LOESS (locally estimated scatterplot smoothing, a nonparametric method that fits smooth curves to data) to explore retention trends over time. To compare interaction rates between feature types, we used Welch’s ANOVA due to violations of normality and homogeneity of variances (Shapiro-Wilk and Levene’s tests, both P<.001). The result was supported by a Kruskal-Wallis test. Post hoc comparisons were conducted using the Games-Howell procedure to account for unequal variances and sample sizes.

**Demand**
To examine engagement trends, we analyzed weekly story views. Pearson correlation and linear regression were used to model the relationship between program week and story views. Residual diagnostics suggested nonlinearity, so we conducted a segmented regression (a regression model that estimates different slopes for separate time intervals) comparing early (Weeks 1-4) and later (Weeks 5-14) phases. Model assumptions were checked through standard diagnostics including Q-Q plots and Breusch-Pagan tests.

**Implementation**
Implementation was quantitatively assessed by analyzing engagement decline over time, operationalized as a reduction in average weekly Instagram story views. Because Instagram does not support direct dropout tracking, we used weekly story view data to calculate relative decreases and visualize cumulative disengagement. This allowed us to examine whether the program delivery maintained user engagement throughout the intervention.

**Practicality**
To assess practicality we examined weekday variation in story views and the relationship between content volume, weekday and retention rate. Mean story views were calculated for each weekday across the intervention. Assumptions for one-way ANOVA were tested: Levene’s test confirmed homogeneity of variances (P=.775), but the Shapiro-Wilk test indicated non-normal residuals (P<.001). Therefore, we applied the Kruskal-Wallis test to compare weekday mean views. To assess whether daily retention varied by content structure, we analyzed two potential influencing factors: the number of story slides and the day of the week. Retention rates were computed as the percentage of viewers who watched the final story slide (excluding post-view entries) relative to those who viewed the first, with values capped at 100%. We used Spearman rank correlation tests to examine nonparametric associations between (1) the number of story slides and retention rate, and (2) weekday (coded 1-7) and retention rate.

**Integration**
To explore integration within school structures, we conducted a descriptive analysis of recruitment-related data, including the number of schools and youth centers contacted, number of information events held, and student participation rates.

**Expansion**To estimate staff resources for recruitment and delivery, we calculated the total time spent on key implementation activities, including institutional outreach, information events, participant coordination, content posting and moderation, and weekly newsletters. Evaluation-related activities were excluded to reflect only the practical requirements for potential scale-up. Total hours were divided by the number of enrolled participants to derive an average staff time per participant.
